# Cognitive mapping: using local knowledge for planning health research

**DOI:** 10.1186/1471-2288-13-96

**Published:** 2013-07-26

**Authors:** Jonathan Stadler, Charles Dugmore, Emilie Venables, Catherine MacPhail, Sinead Delany-Moretlwe

**Affiliations:** 1 Wits Reproductive Health and HIV Institute, School of Clinical Medicine, Witwatersrand University, PO Box 18512, Hillbrow, Johannesburg, 2038, South Africa; 2 Witwatersrand University History Workshop, 1 Jan Smuts Avenue, Braamfontein, Johannesburg, 2000, South Africa

**Keywords:** Cognitive mapping, Clinical trials, HIV prevention, South Africa, Hillbrow, Orange farm, Community

## Abstract

**Background:**

Cognitive mapping is a participatory research methodology that documents, in visual form, a construct of the local environment in which people live and work. We adapted this method to provide detailed data about study locales to inform recruitment and retention strategies for HIV prevention community based clinical trials.

**Methods:**

Four cognitive mapping studies were undertaken between 2005 and 2010, in and around Johannesburg in Orange Farm, and Hillbrow. Participants included members of clinical trial Community Advisory Boards (CAB), young people recruited from schools in Hillbrow and an organization for out of school youth, and men recruited from a database of men in the community interested in taking part in research. These groups participated in reconnaissance walks and in depth interviews, and drew detailed sketch maps.

**Results:**

The cognitive maps defined the physical boundaries of the research locales, provided insights into their social histories, and identified important characteristics of the population such as movement, social and sexual networks, ethnic and other divisions. Important differences between the official cartographic maps and the cognitive maps were raised. The mapping data was applied by identifying key areas for recruitment that recruitment staff members were less familiar with and that may otherwise have been overlooked.

**Conclusions:**

Cognitive mapping is an effective, rapid and low cost method that can be used to inform recruitment and retention strategies for community-based clinical trial research. The method also provides a means for clinical trial researchers to involve the local community in research and to familiarise them with the social setting.

## Background

The capacity of community based health research to assess the efficacy of novel interventions to prevent the spread of HIV depends on the enrolment of sufficient numbers of participants who fit the study inclusion criteria and will remain in the study throughout its duration. Failure to retain high percentages of trial participants due to loss to follow up may undermine the outcomes of the trial [1]. Barriers to recruitment include the diversity of populations, language and cultural differences, as well as local narratives about medical research that emphasise its dangers [2]. Therefore, recruitment and retention are critical issues to consider in the planning stages of health research. Most clinical trials follow standardised protocols that are implemented in diverse social contexts; it follows that recruitment and retention strategies need to be tailored to different settings [3]. Detailed information about the research context can greatly assist clinical trial managers in enhancing recruitment plans.

Since the early 2000s, the Wits Reproductive Health and HIV Institute (WRHI) embarked on community-based HIV prevention studies in the greater Johannesburg area and inner city. This includes, amongst others, research on microbicides, herpes suppressive therapy, HIV testing and a vaccine feasibility study. The studies involve HIV negative and positive women, men, youth and HIV sero-discordant couples. As a first step in preparing for the research, data is collected about the population characteristics, community resources and structures and local organisations. Clinical trialists use this information to engage with community structures about the proposed research and establish community advisory boards (CAB).

It is also critical to develop a local view of the boundaries of the study area, identify areas for recruitment and information dissemination. Ethnographic research utilising participant observation techniques is the ideal methodology for generating a nuanced understanding the local context, but this requires an investment in time and personnel that is not always feasible in the start-up stages of a research project [4]. To address this need for contextual understanding under the constraints of a clinical trial we adapted the cognitive mapping approach.

Mapping is a powerful research tool and the importance of spatial dynamics in shaping health is well known to epidemiologists and social scientists [5]. Different applications of mapping methodologies can inform the targeting of prevention programs and information dissemination. For example, the PLACE methodology assists in planning HIV prevention activities by identifying the social spaces where people meet new sexual partners. Exploring the spatial dynamics of HIV spread focuses on risky places instead of risk behaviours [6], and highlights the role of locale in shaping risk [7]. Cravey *et al.*[8] outline the application of mapping methodologies to describe ‘social spatial knowledge networks’ for sharing and disseminating public health information.

In addressing our particular needs for insights into the local setting of health research and in particular, clinical trials, the application of the cognitive mapping method appeared to hold promise. Cognitive mapping differs from standardised community mapping. It focuses on local or *emic* knowledge and produces several maps that may differ vastly from official maps. Moreover, cognitive maps reflect upon the social and historical dimensions of a locale; particular points in the landscape contain stories of events and contribute toward a meta-narrative of the community. Cognitive mapping does not only describe spatial arrangements but reveals how people think about and use space.

Kevin Lynch, an urban planner, first developed the cognitive mapping approach to mapping urban environments in the 1960s. Lynch focussed on those features of the environment that people negotiated as they moved around their cities. He studied Boston, Los Angeles and New Jersey in the US with small samples of residents, and asked them to describe how they moved through their surroundings and how they would direct a stranger around the same environment. Lynch suggested that a collective or public ‘image’ of the city could be constructed composed of three components: identity, structure and meaning [9]. Since Lynch’s initial work, cognitive mapping has been applied to disciplines such as psychology [10] and education [11]. It has also been applied, albeit in a limited fashion, to health care settings; one study analysed the orientation and flow of people in a hospital building [12].

The process of cognitive mapping is meant to be highly participatory. As we describe later in the article, community members are engaged in drawing maps, guiding the researchers and discussing the research outcomes. The participatory nature of the method presents opportunities to answer the call for greater community participation and dialogue more generally in health research, [13,14], and clinical trials [15,16].

The article describes the application of cognitive mapping using four case studies in two populations, identifies the key findings and modifications that were made to the method, and makes suggestions about the application of cognitive mapping in clinical trial research.

## Methods

Four cognitive mapping researches were undertaken between 2005 and 2010, in and around Johannesburg in Orange Farm and Hillbrow. Participants included members of the WRHI clinical trial Community Advisory Boards (CAB) representing community interests in the research studies, young people from schools in Hillbrow and an organization for out of school youth, and men resident in Hillbrow. Each study involved between eleven and 20 participants. Three of the four cognitive maps were developed using conventional methods and, in one, participatory photography was used as an additional data collection tool.

### Selection and recruitment of research participants

#### Community Advisory Boards (CAB)

The WRHI has a long-term relationship with the Orange Farm and Hillbrow CAB, dating back to 2000. These groups, consisting of adult men and women provide advice and guidance on research conducted by the WRHI. We presented the project to the CAB members at a regular meeting and invited members to participate in the research.

#### Men

Male participants were chosen from a database of men in the Hillbrow community who had expressed interest in being involved in HIV prevention research. They were then contacted by the community outreach team and asked if they would like to participate in the project.

#### Youth

We divided participants into in-and out-of school youth. For the in-school youth we approached two local Hillbrow high schools with which we had a relationship and asked for volunteers aged between 15–18. For the older age group (18–25 years) we asked for volunteers from a local community-based organization.

Research participants signed informed consent before taking part in the cognitive mapping research. Assent was obtained from participants who were under 18 years, after documenting parent consent. The University of the Witwatersrand Human Research Ethics Committee approved the research (Table 1).

**Table 1:** Description of the four cognitive mapping exercises

**Project name**	**Date**	**Participants**	**Number**	**Maps**
Hillbrow CAB	May-July 2004	Hillbrow CAB members	12	2
Orange Farm CAB	March 2005	Orange Farm CAB members	15	5
Youth VCT	June-September 2005	Hillbrow Youth	12	4
Visual Hillbrow (Men)	April 2010	Hillbrow Men	11	9
Totals			50	50

### Specific research tools

A cognitive mapping manual was developed to guide the research teams [17]. Each project built on the experiences of previous research, and adapted the methodological tools to their particular research objectives, and each study offers a different experience of the same methodology. The most recent adaption included photography in addition to map drawing.

The eleven participants of ‘Visual Hillbrow’ took part in a four-day participatory photography project. They were taught basic photography skills that allowed them to document their experiences of Hillbrow. Men were asked to focus on the (un) healthy spaces of their environment as well as ‘male’ spaces within Hillbrow. Detailed findings and photographs are published elsewhere [18].

### The research settings

**Figure 1:**
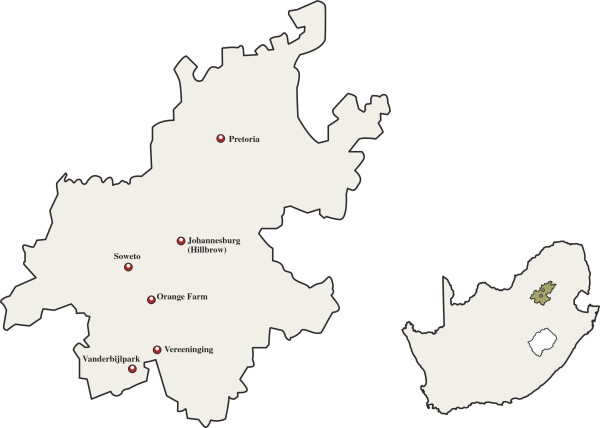
Gauteng Province, South Africa, showing the location of Orange Farm and Hillbrow in relation to nearby towns and cities.

The cognitive mapping activities took place in Hillbrow and Orange Farm, located within the greater Johannesburg area (See Figure 1). These locations are the sites of on-going clinical trials and other research activities of the WRHI, and the cognitive mapping research informed the broader research agenda. For our research purposes these locations create an interesting contrast between densely populated inner city suburbs (Hillbrow), and the unplanned, ‘informal’ settings (Orange Farm), representing the broad spread of residential settlements types in South Africa.

### Hillbrow

Hillbrow was established in the late 19th Century as an upscale residential area consisting only of detached houses; neither shops nor ‘canteens’ were originally allowed in this ‘healthiest and most fashionable suburb of Johannesburg’ [19]. Following the building boom of 1965–75 Hillbrow became a prime location for transients [20], a feature that contributed to Hillbrow’s character as a place for immigrants and young people. By the 1980s, population densities in the suburb had increased to 180 persons per acre; far more than the envisaged 40 per acre predicted in 1946 by local authorities. Hillbrow became ‘…a high density, high-rise, inner-city neighbourhood’ [19].

Currently, over 50 000 people live in approximately 200 high rise apartment buildings and hotels spread over one square kilometre. The suburb has a large population of immigrants and economic migrants from sub-Saharan and West Africa as well as from within South Africa. Recent estimates are that half of the residents of Hillbrow are foreign-born, young and about two thirds are males [21]. The suburb has a large population of between 5 000 and 10 000 sex workers who work from the streets and numerous brothels in former hotels and apartments [22].

### Orange farm

Orange Farm, situated 40 kilometres to the south west of the Johannesburg inner city, emerged as a definable community in 1988 when a number of impoverished residents from Soweto (the South Western Townships, located to the south of the Johannesburg inner city) began to build informal houses on farmland. Many of the early residents were tenants and sub-tenants who lived in the crowded backyards of Soweto homes, renting cramped structures such as prefabricated sheds referred to as chicken coops (*Mkhukhu*) or *Zozo* (wooden shed). In Orange Farm, settlers could own their own land and were soon joined by homeless people from the industrial towns of Vereeniging and Vanderbijlpark. Orange Farm grew very rapidly and soon a number of extensions were added. While about two-thirds of the housing stock in Orange Farm is informal, a large number of brick houses have been built in the more established areas.

Orange Farm has undergone significant growth and is today a peri-urban settlement of approximately 170 000 people. Many new arrivals are attracted by the desire to find work; however employment is scarce and transport costs are high. The state provides government housing for many residents and electricity for most households. Water is a scarce resource and limited access is through pre-paid services, a source of considerable political tension. Major development projects are implemented in Orange Farm: a multipurpose community centre, which includes an Olympic-size swimming pool, an information centre with Internet access, and the Nike Chris Hani Sports Complex. However, many residents depend on minimal incomes [23].

### The process of cognitive mapping

Cognitive mapping is a fundamental human ability to comprehend the world through the production of representations or ‘maps in the mind’. It is a mental engagement with the spatial environment that enables people to analyse, interpret, and use information about their surroundings [24]. Lynch [9] investigated the ‘legibility’ of the ‘cityscape’ by identifying five key ‘elements’ that people use to construct their ‘mental maps’ of their urban environments: paths, edges, districts, nodes and landmarks:

•*Paths* are channels through which the city dweller moves from point of origin to point of destination e.g. streets or walkways;

•*Edges* are boundaries like walls, railway lines, highways and other barriers that mark off a distinct area or district;

•*Districts* are distinct areas with a common, identifying character;

•*Nodes* are key junctions like a bus stop or railway terminus or an area where people cluster like a shopping centre or a taxi rank;

•Finally, *landmarks* are usually striking physical objects such as towers that city dwellers use as reference points to orient themselves.

The intensity of each of these elements will vary from individual to individual but cumulatively these can be compared and superimposed to construct a ‘public image’ of the built environment that is held in common by a specific community [9].

Lynch’s methodology requires an initial investigation of the terrain through conventional means of a cartographic map, analysis of secondary literature and ‘field reconnaissance’ in the form of a transect walk or drive through. Researchers trained in Lynch’s methods take detailed notes and produce a map that represents the landmarks, districts, nodes, paths and edges using symbols.

The second stage of research requires in-depth, semi-structured interviews with a manageable sample of residents who answer set questions designed by Lynch to elicit each individual’s unique mental image of their surroundings. For example, they are required to describe in detail what they would expect to see as they move from point A to point B in the locale and how they would direct a stranger to make the same journey. Finally, participants are asked to sketch their locale while the researchers take careful note of the sequence in which the different components are sketched.

Our first two cognitive mapping exercises followed this methodology fairly strictly with participants drawn from the CABs in Orange Farm and Hillbrow. Following the analysis of the data we applied the same method but focussed on youth in Hillbrow to get a different perspective. The fourth mapping exercise was undertaken with men in Hillbrow.

### Data analysis

Data collected in the cognitive mapping research include participant and researcher produced sketch maps, ethnographic field-notes, in-depth interview transcripts, notes from participant observation and photographs. All interviews were recorded and transcribed. Those that were conducted in SeSotho or IsiZulu (the two dominant languages) were translated into English.

The first step in data analysis was to transcribe the information contained within the maps into tables, to enumerate the occurrence of particular features. The next step was to draw composite maps based on the official, researcher, and participant maps. Field notes from the discussions and interviews were reviewed and salient themes noted as well as divergences and discrepancies between the different maps. This information was collated, and tabulated and transformed into a ‘public image’ that forms the basis of a consolidated cognitive map. These maps are then compared and analysed to produce an explanation of the similarities and differences.

## Results

Here we present the results from the research activities conducted in Orange Farm and Hillbrow and draw attention to specific examples that illustrate the use of the method.

### Reconnaissance walk

The reconnaissance walk is an opportunity for researchers and research participants to reflect on their surroundings, to ask questions and engage with each other in an open-ended fashion, allowing for wide ranging discussion. Field researchers, trained in identifying Lynch’s five elements described above, assessed the degree of visibility of these elements while walking around the locale. They noted direction, topography, what was visible at various points, the sounds, smells, and feelings. This process, described as the construction of ‘place inventories’, included a description of the quality of houses and buildings, the identification of open spaces where outsiders could become lost, possible ‘confused’ or ‘blurred’ points and ‘blank spots’, that is, areas that were too undifferentiated and bland to be remembered clearly. Landmarks were also identified and our informants explained their significance. The reconnaissance walk noted the edges, nodes, paths and districts in each locale. This information was used to create a map layered on top of a standard cartographic map.

In Visual Hillbrow, members of the research team conducted a series of shorter reconnaissance walks before the participants were recruited, and then accompanied the participants during the photo project, taking notes in addition to the visual data that was being collected. Due to the size of Orange Farm, the reconnaissance walks were conducted over several days. Members of the CAB from Orange Farm narrated the local history, physical features and landmarks and paths to the researchers. In Hillbrow, the initial reconnaissance walk was undertaken over one day by the researchers together with a staff member who had long term experience of working in Hillbrow. In addition, the researchers drove along the same route at night (walking was inadvisable due to the high levels of crime), taking notes and photographs.

### Sketch maps and interviews

The sketch maps produced by our informants and the interviews we held with them represent another layer of information. In the following we summarise the findings from this data following Lynch’s five elements, and raise the most salient themes.

### Paths

**Figure 2:**
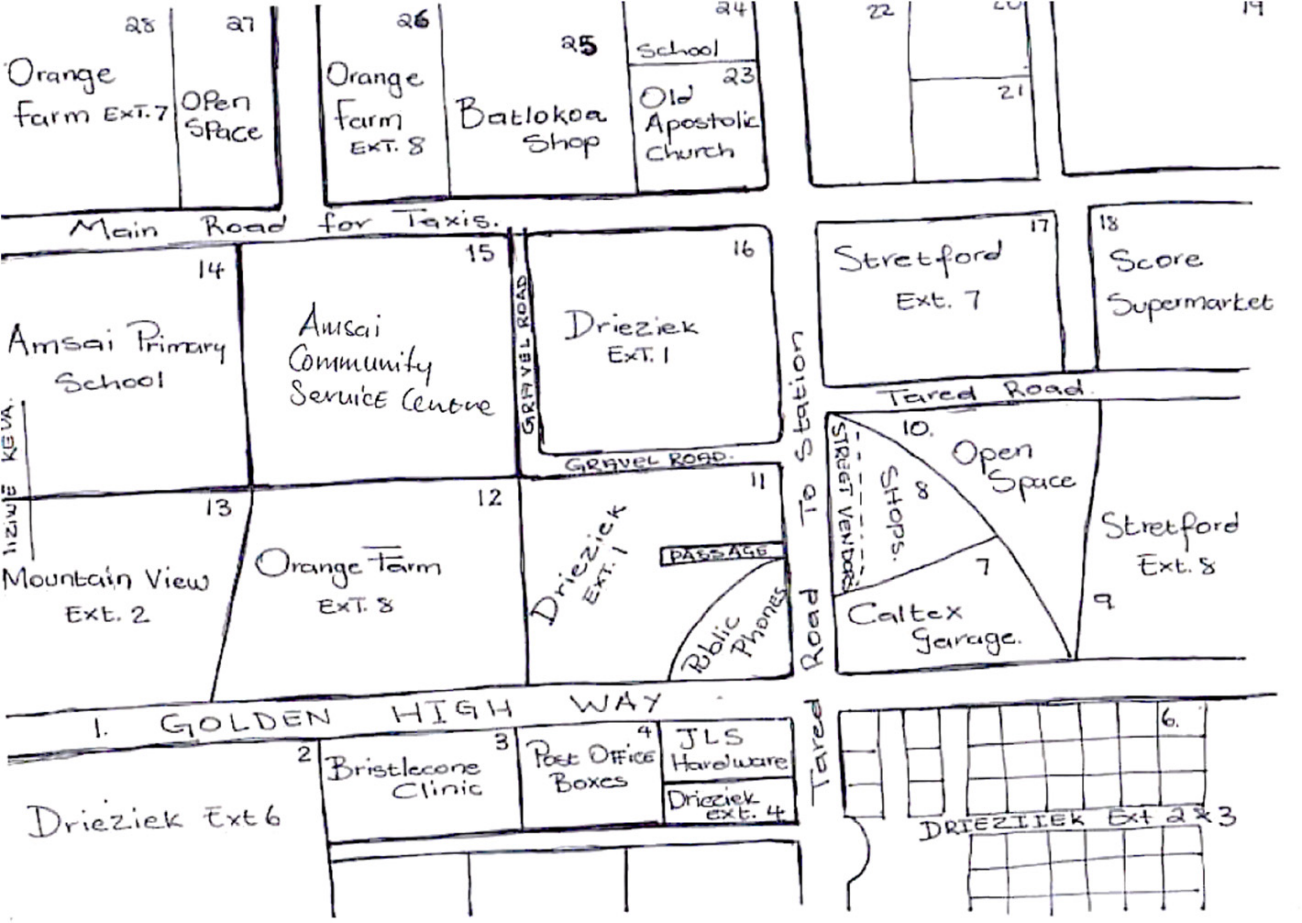
Sketch Map of Orange Farm, drawn by an Orange Farm CAB member.

The Orange Farm maps accurately depicted and named the main roads as well as uncovered gravel roads despite the absence of road markings (Figure 2). Another notable feature of the Orange Farm sketches is that the paths extend to the edge of the page; there are few edges, particularly in the northern most areas of the map. This is significant: the paths represent conduits to Soweto and Greater Johannesburg, places where many Orange Farmers seek work and where many hail from and return to visit kinsfolk and friends. These areas are seamlessly integrated into Orange Farmers’ sense of place.

**Figure 3:**
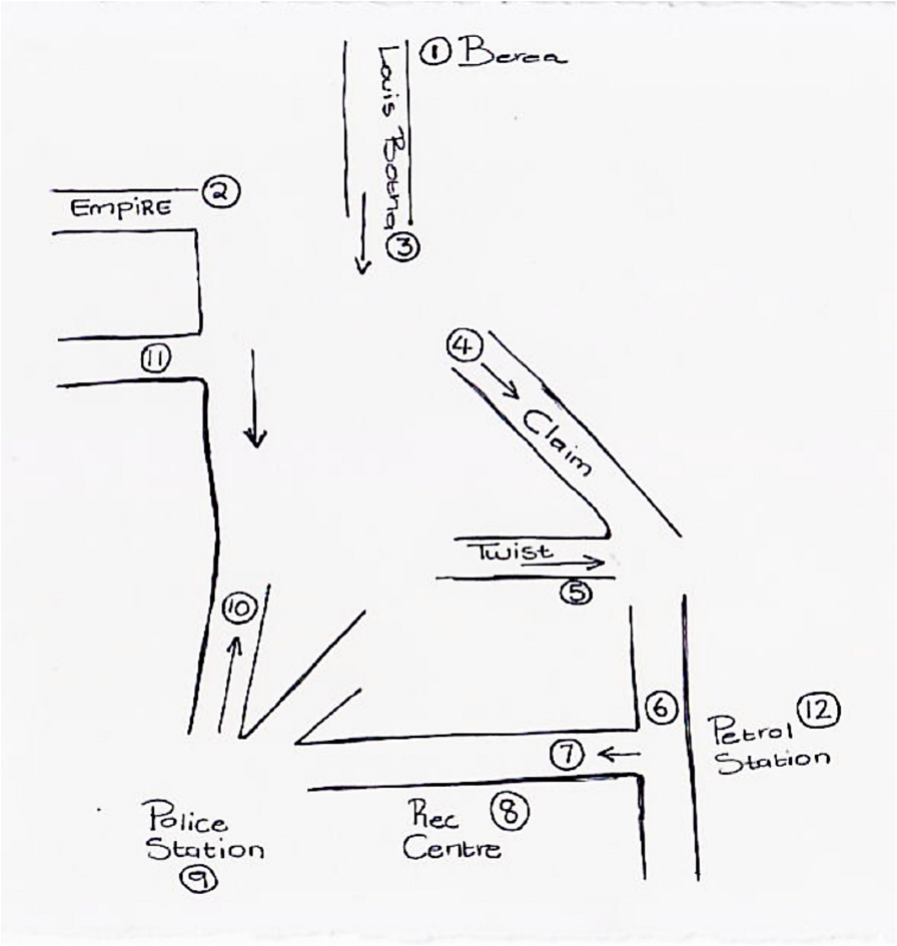
Sketch Map of Hillbrow, drawn by a Hillbrow CAB member.

In contrast, the Hillbrow sketch maps created by the Community Advisory Board (CAB) members depict movement *through* the area; for instance many of the sketch maps of Hillbrow use arrows to indicate pathways in and out of the area, sometimes with very little other detail (Figure 3). The map highlights movement, reinforced by informants’ description of Hillbrow as a ‘very fast place’. Although many of the CAB members live in Hillbrow, their sketch maps do not suggest a strong emotional attachment to the area. The overriding impression we gleaned from our conversations was that residents leave their apartments hastily in the morning and return with equal haste into the relative safety of the building at night. Risk and danger was a prominent theme in discussions with our CAB informants. Many could recount being victims of crime while moving through Hillbrow. A CAB member was mugged for her bag at a particular intersection; another commented that he would not use a certain street ‘because there are many people hanging around most of the time’.

**Figure 4:**
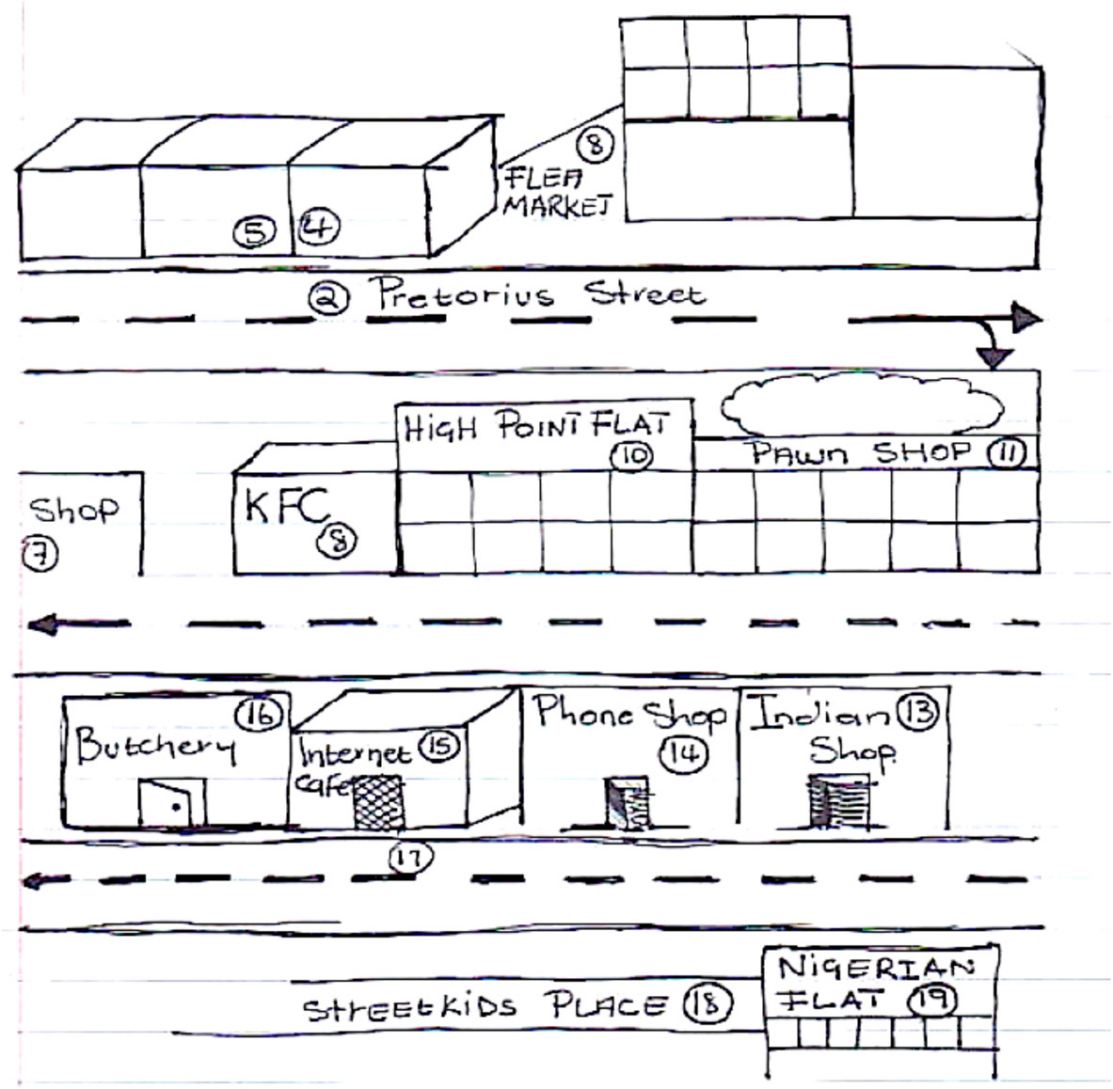
Sketch Map of Hillbrow, drawn by a Youth group member.

**Figure 5:**
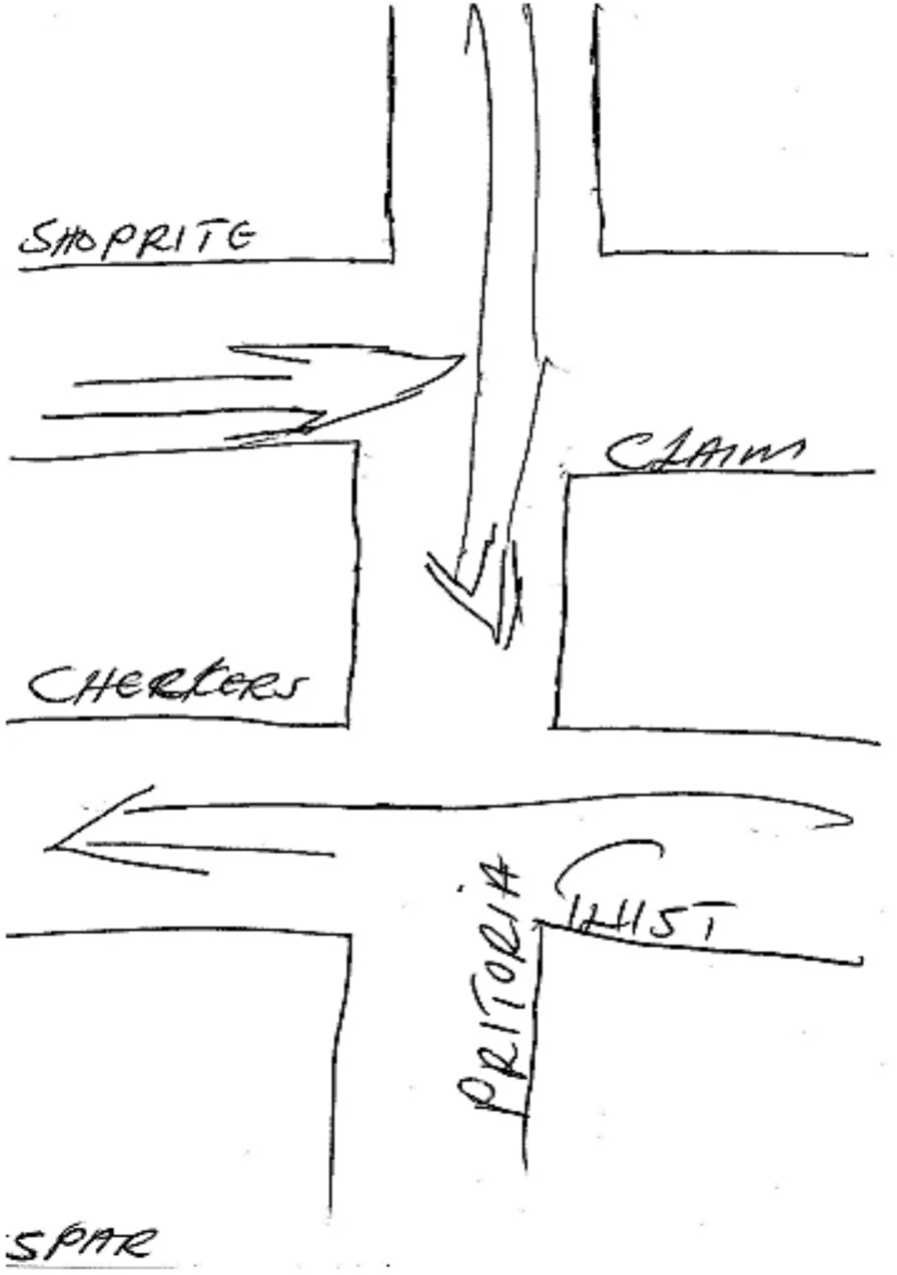
Sketch Map of Hillbrow, drawn by a Men’s group member.

In contrast to the sketch maps produced by the CAB (above), those drawn by the youth were far more detailed and reflect a greater sense of engagement with the locale (Figure 4). Yet they too talked about Hillbrow as being a very dangerous place for children. Similarly, men who participated in the Visual Hillbrow exercise talked about quickly moving through the area due to feeling unsafe. A sketch map (Figure 5) drawn by a member of the men’s group is remarkably similar to the CAB sketch map (Figure 3), consisting mainly of hastily drawn arrows, highlighting the rapid movement through the locale.

### Edges

**Figure 6:**
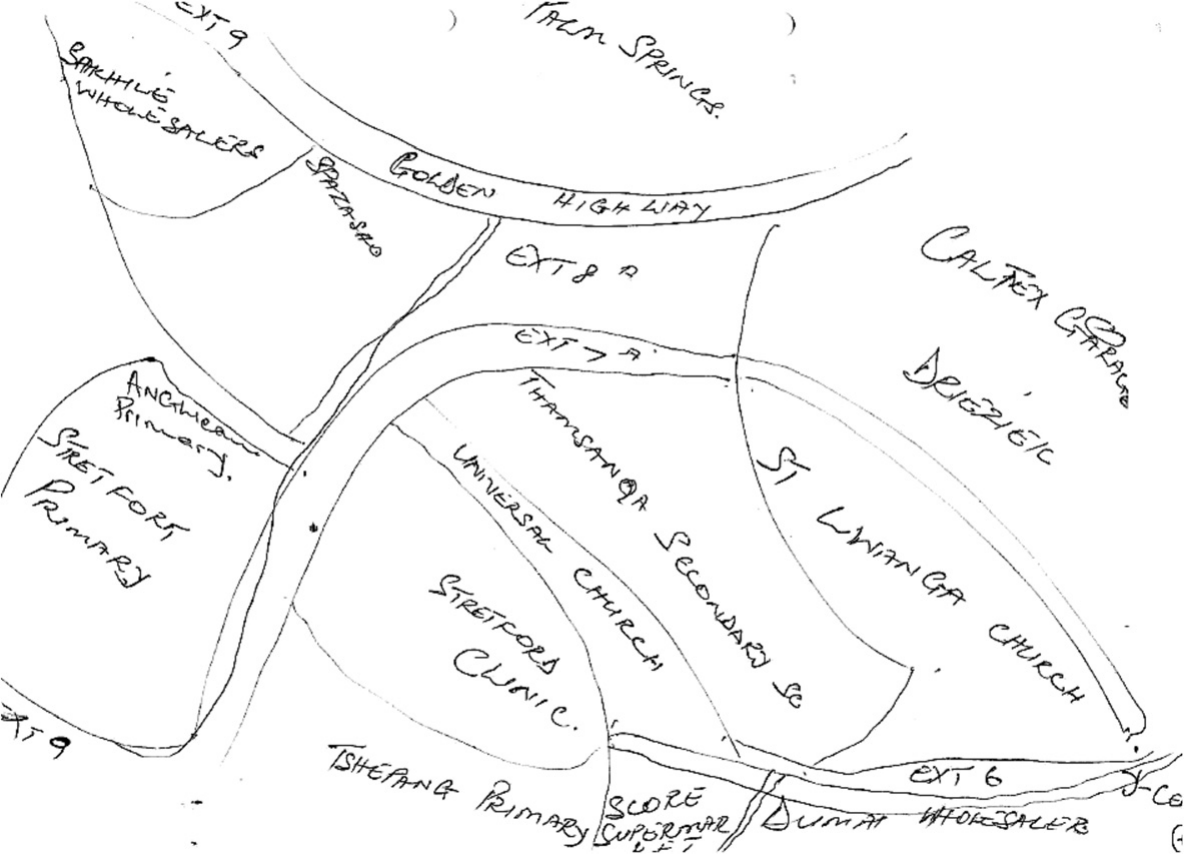
Sketch Map of Orange Farm, drawn by an Orange Farm CAB member.

The edges portrayed in the sketch maps reveal how residents think about their social orientation in relation to adjacent communities. Noticeably, the sketch maps produced of Orange Farm extends the borders of the official map to include adjacent areas, despite the existence of clear physical borders such as roads. For instance, Palm Springs is a separate township from Orange Farm, physically cut off from by a busy main road (Figure 6). As a planned residential area with water, sanitation and electricity and tarred streets, it contrasts with the chaotic appearance of Orange Farm. The majority of residential units in Palm Springs are ‘bonded houses’, purchased through bank loans. Yet, Palm Springs is clearly included as part of Orange Farm in sketch maps (Figure 6).

In the CAB and youth group sketch maps, many borders of the Hillbrow locale were identified as ‘confused’. In one sketch map the informant has written the words ‘Do not know this side’. The proliferation of confused areas also reflects negative sentiments associated with adjoining neighbourhoods. In the notes accompanying sketches a youth group member said, ‘I am sorry I do not know more about the area … but I do not feel safe in this environment’. Others were more explicit; for example, one of the youth noted: ‘I see people sitting around until the sun goes down and there is nothing to do…you see buildings [have] water everywhere and it smells’. The same respondent also said that ‘the way it’s dirty…full of foreigners…I see guys smoking *dagga* [marijuana] and [inhaling] glue, and many people are not working’. Others made reference to the presence of Nigerian immigrants in the area: ‘The building is surrounded by many Nigerian guys, day and night. It is a place where thugs like to hang around, where one can get mugged’.

### Districts

Although the Orange Farm and Hillbrow sketch maps identified districts, the sketches produced of the two locales did not give equal weight to these divisions.

**Figure 7:**
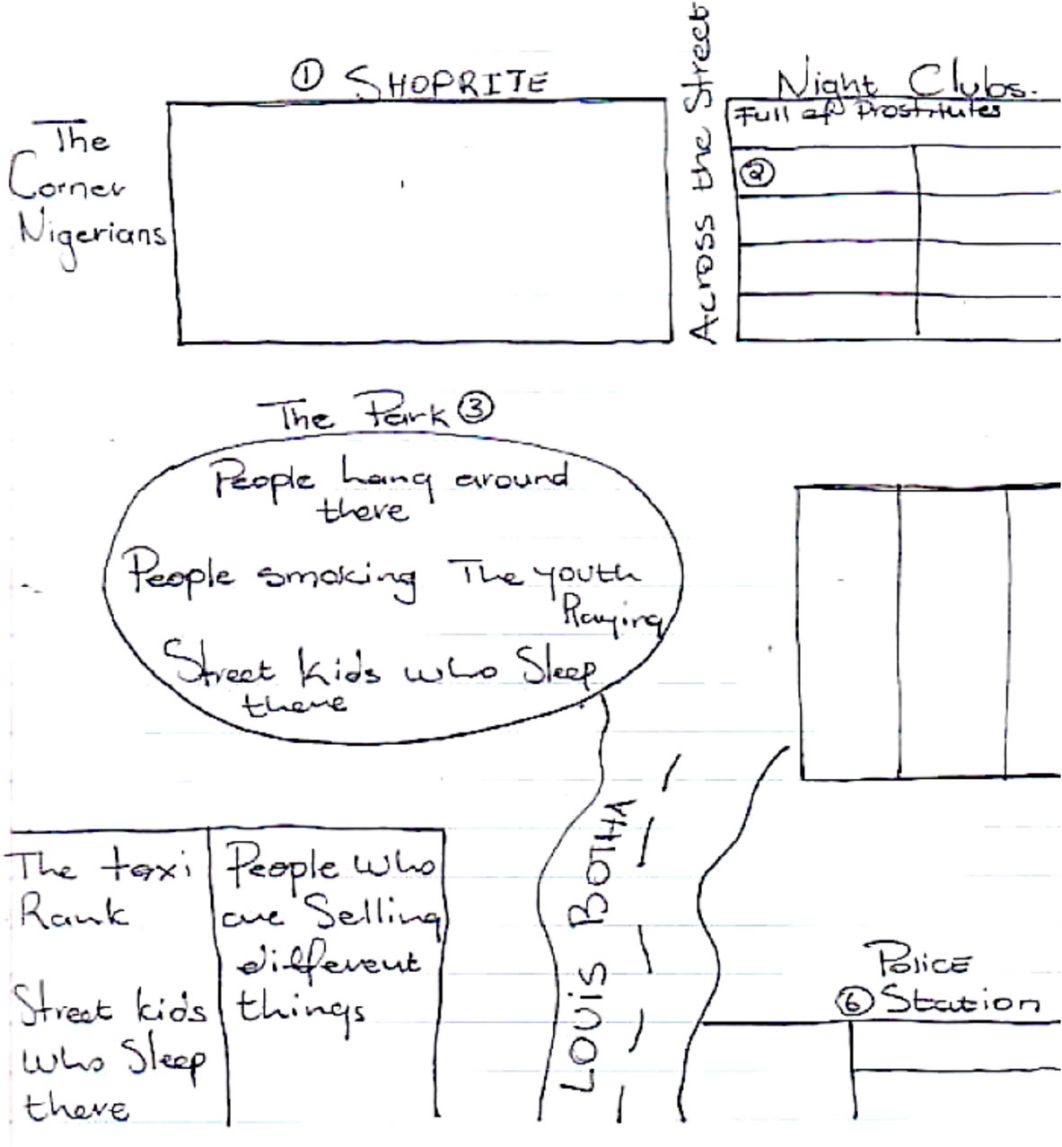
Sketch Map of Hillbrow, drawn by a Youth group member.

The sketch maps of Hillbrow distinguished between residential and business districts, drawing attention to shops and fast food outlets. Equally prominent were districts defined by ethnicity. For example, two neighbourhoods are identified as ‘Little Lagos’ because of the presence of Nigerian immigrants. The sketch map produced by a member of the youth group, details an area as ‘Nigerian’, and a shop is identified as belonging to an ‘Indian’. Another sketch from the same group identifies the ‘[street] corner Nigerians’ (Figure 4). These areas were associated with the illegal drug trade and sex work. A youth group member included the comments in his sketch map: ‘prostitutes, people who smoke, people selling, a mix of different people, street kids’ (Figure 7). In contrast, the Orange Farm sketch maps identified different districts, corresponding to the official divisions within the township, yet the social identity of these districts were played down and made less obvious in the sketches (Figures 2 and 6).

### Nodes

The sketch maps identified nodes where people socialise and gather. While our informants in Orange Farm and Hillbrow identified several key nodes, for some, these locales were almost node free.

The sketch maps of Orange Farm identify transport nodes, mainly taxi ranks and the railway station. Orange Farm is an isolated community with limited resources and transport junctions are important to its residents employed in Johannesburg. Transport nodes are also meeting places regularly visited by residents. Other busy areas such as a roving pension day market were also important nodes (Figures 2 and 6).

**Figure 8:**
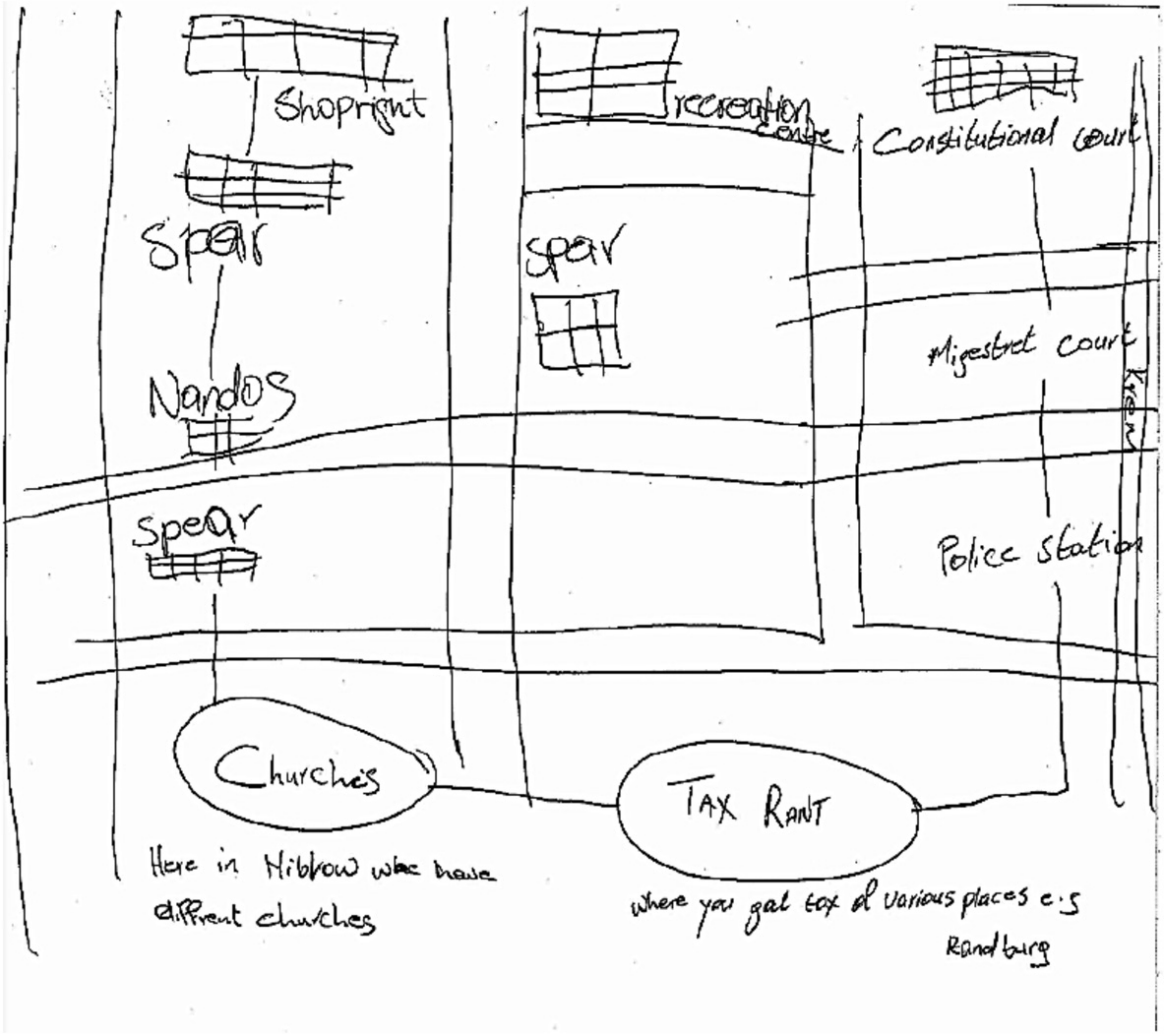
Sketch Map of Hillbrow, drawn by a Men’s group member.

Similarly, the Hillbrow CAB sketch maps identified taxi ranks as nodes where people gather. Equally important were public recreational areas: men identified parks, bars and clubs as key nodes, although they often lacked of personal experience of these nodes, talking of these as places where they ‘used to go’ or ‘others go’, rather than nodes that they currently frequented. In one map, drawn by a man from the Men’s Group, specific mention is made of churches and the taxi rank, as well as various fast food outlets and shops (See Figure 8).

For many youth Hillbrow was remarkably node free. They spoke of their fears of public spaces although their maps identified fast food restaurants and super markets and recreational centres (the theatre and a park) as nodes where young people hang out. Similar to our male informants, they expressed limited personal experience of these nodes, often due to concerns about personal safety. A male youth noted: ‘ya, well what I can say, is that Hillbrow is not a place where you can socialise. On my side I think I’m scared of that place. I lived a long time in Hillbrow and I think it gets worse day by day’.

### Landmarks

In the Hillbrow sketch maps, fast food restaurants and shops were more popular landmarks, particularly for the youth. Given that people mentioned violence, crime and danger as common experiences of life in Hillbrow, the police station was unsurprisingly significant as a landmark in multiple sketches (Figure 7). In Orange Farm, research participants identified public buildings and schools as important landmarks (See Figures 2 and 6).

### The cognitive maps

**Figure 9:**
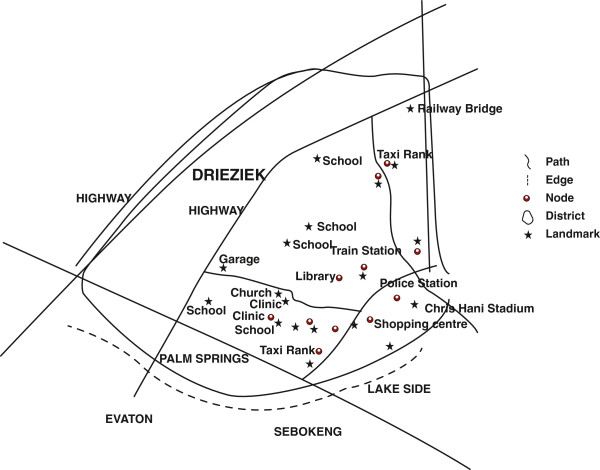
Cognitive Map of Orange Farm.

**Figure 10:**
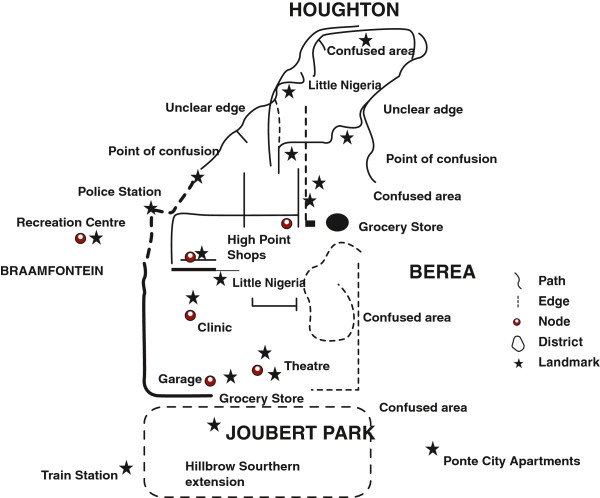
Cognitive Map of Hillbrow.

The Cognitive Maps we developed draw on multiple sets of data: the sketch maps produced by our research participants, our observations and commentary from reconnaissance walks, photographs, information from interviews, and the official cartographic maps of the areas. We produced two Cognitive Maps, one for each location (Figures 9 and 10).

Of particular interest are the discrepancies between the official, the researcher, and emic or insider representations of the locales, reflected in the cognitive maps. Cognitive maps are invariably incomplete and distorted; these distortions and omissions are important as they reflect peoples’ experiences of the places where they live and work. In both Orange Farm and Hillbrow, the borders depicted in informants’ sketch maps differed considerably from those laid down by official cartographic maps.

The cartographic map of Orange Farm identifies eight administrative ‘sections’. Our participants identified these sections with ease, even when these did not correspond with any visible elements. For example, they pointed out where an administrative border that cut through a residential stand. They also noted that more recent arrivals in Orange Farm live in newly released areas and set up temporary shacks, distinct from the older residents who live in brick houses. Yet, despite their knowledge of these official boundaries, our participants downplayed the divisions between districts and instead referred to Orange Farm as a whole, highlighting the unity of the locale. However, neither these divisions were not reflected in their sketch maps.

The cognitive map also reflects the expansion of Orange Farm to include areas that are not officially recognised as included in the locale. For example, we assumed that Lake Side was a distinct neighbourhood. The homes in Lake Side are uniform and arranged neatly unlike the haphazard lay out of the neighbourhoods of Orange Farm where housing types range from tin shacks to brick townhouses. Property developers established Lake Side for professional salaried individuals. Yet, our informants pointed out important social and economic relationships linking individuals who resided on either side of the main road. Astutely, they noted the sexual relationships between wealthier men residing in neighbouring suburbs and young unmarried women who hailed from Orange Farm (Figure 9).

The cognitive map produced for Hillbrow also differs considerably with the official representation of the suburb in cartographic maps. The entire suburb was reoriented to include areas to the south and east, while neglecting the areas in the northern most area (Figure 10). Part of the reason for this is the presence of a major transport node in the southeast where commuter taxis collect and discharge passengers from Hillbrow. However, the exclusion of the northern section of Hillbrow also reflects the ineligibility of the northern-most boundaries of Hillbrow that borders on more affluent suburbs and does not fit in with the overall image of the area.

The cognitive maps also reflect the differences between official and local ideas about landmarks. For instance, the post office tower is the most prominent feature in Hillbrow, often displayed in tourist brochures of Johannesburg, yet was excluded from the cognitive map. In the Orange Farm cognitive map, considerable emphasis is placed on local landmarks that are meaningful for peoples’ sense of history and identity. Certain landmarks such as schools were important markers of identity and a source of pride, indicating the high value placed on educational progress. Another major landmark was The *Chris Hani / Nike Sports Stadium*, named after the late leader of the South African Communist Party (SACP), often evoking strong emotions. Chris Hani adopted Orange Farm as ‘Palestine’, symbolically aligning the struggle of early settlers in Orange Farm for land with that of the Palestinian people.

The numerous paths revealed by the cognitive maps of Orange Farm reveal an insider’s social orientation and experiences of living and working in the locales. Of particular interest is the inclusion and omission of certain roads and walkways; this describes peoples’ movement and their use and avoidance of particular spaces. The care given to these internal, unmarked dust roads in contrast to the main roads suggests a strong local orientation. As people travel along these paths within the locale they meet and exchange information, reasserting social relations. In this way, the paths are ties that bind rather than arteries through which people move, creating opportunities for sociability.

## Discussion

Cognitive mapping is a methodology that can be rapidly implemented that offers detailed insights into a particular locale in preparation for clinical trial research. Cognitive mapping served multiple purposes in the start-up phase of clinical trials run by the WRHI and greatly enhanced our knowledge of the communities where we work. It is able to do so because it works with a small sample of residents or particular interest groups. In our application of the method we worked with the CAB from Orange Farm and Hillbrow and later included youth and men in similar exercises. In total we worked with 50 individuals in two geographic areas and produced two cognitive maps. The method was relatively easy to implement by fieldworkers with the guidance of a social scientist.

As with most qualitative research methods, the open-ended style of cognitive mapping avoids researcher preconceptions and bias. For example, asking our informants to draw a sketch map opened up discussions about danger and safety without probing. The sketch maps also stimulated questions about missing details; the ‘blank spots’ and unclear areas that indicated uncertainties and concerns with specific areas. In this way cognitive mapping avoids predictable responses and provides access to a highly personal and intimate perception of the built environment that more conventional techniques fail to produce.

### Applying cognitive mapping to clinical trials

The cognitive mapping tools, notably the reconnaissance walks, created opportunities for the researchers to become familiar with locales in which they were expected to start recruiting participants for clinical trials. In dense urban settlements with reputations for violence and crime (such as Hillbrow), the research exercises increased staff confidence to move around the locale. Moreover, the maps and discussions about safety identified where recruitment could take place and where it should be avoided. Most importantly the method compelled staff to consider recruitment nodes beyond the standard health care settings such as clinics and hospitals. The maps also identified areas that may have been overlooked. For example, the cognitive map of Orange Farm and Hillbrow extended the recruitment area to include locales beyond official borders.

Insights gleaned from the analysis of the maps provided important information about the social context of the research settings and helped to formulate strategies for recruitment and retention. For example, Hillbrow is a transport hub meaning that our recruitment activities would reach a larger pool of potential participants many of whom may only pass through Hillbrow on their way to other destinations. Many of the people volunteering for the trials would reside outside of Hillbrow, but would access the trial clinic by using public transport.

Including different categories of resident (in the Hillbrow mapping research) highlights the importance of developing different strategies for different populations. For example, there are limited informal recruitment sites and schools and social organizations remain the best option for recruiting young people into research studies. Men’s involvement enabled the research team to learn more about men’s experiences of living in Hillbrow, and gain insight into potential recruitment sites in the area for future studies and clinical trials involving men.

The cognitive mapping exercise also provided insights into the representivity of our CAB members. The Hillbrow CAB members who participated in the mapping exercises produced sketch maps that revealed their limited spatial orientation mostly describing routes in and out of the suburb. In contrast, the sketch maps from the Orange Farm CAB were far more detailed and covered a greater area, reflecting a higher level of involvement in their community.

The maps had additional practical applications. Due to the unplanned nature of Orange Farm, community recruiters often struggle to identify the location of participants’ dwellings. The maps were used to identify landmarks that served as triangulation points to determine where research participants live when confirming locator information.

Finally, cognitive mapping promotes participation in research. Our informants acted as guides during the reconnaissance walks and were responsible for producing the research materials such as sketch maps and photographs.

### Defining ‘the community’

Definitions of whom and what constitutes ‘community’ frequently assume shared space and social cohesiveness and bounded-ness. Yet, the boundaries of communities are symbolic and only exist in so far as people believe in them [25]. This raises questions of how to define the unit of study for clinical trial research, but also of what constitutes ‘community’ representation [26] particularly with regard to community advisory boards.

Cognitive mapping research offers insights into the spatial and social constructs of community. Cognitive maps challenge the official, external views of the spatial orientation of the research sites, and redefine the boundaries. They show how constructs of what constitutes the ‘community’ are often at odds with local constructs. This corresponds to the anthropological opposition between *emic* (insider) and *etic* (external) perspectives. Cognitive maps provide a means of accessing emic constructs and spatial constructs.

The case studies of Orange Farm and Hillbrow presented in this paper reveal distinctive forms of community identity. Residents of Orange Farm identify strongly with the locale in which they live and exhibit unified and closely networked social ties that cut across ethnicity, origin and social class. Although many of the current residents of Orange Farm are recent arrivals, our informants’ commentary on the cognitive maps expressed a strong sense of emotional attachment through reference to historical events. This contributes toward the construction of a common social identity as Orange Farmers.

Hillbrow, in contrast, shares little of these sentiments, with noticeable divisions between neighbourhoods and an absence of safe shared public spaces. Although Hillbrow has a long history dating back to the turn of the 20th century, its residents’ history is very recent due in part to the rapid changes that have taken place in the suburb and because many residents are recent arrivals. A sense of historical continuity is lacking, and the various landmarks that may have been applicable a few years previously have little meaning for current residents. Residents have very limited ‘pictures’ of Hillbrow in their minds and excluded certain areas altogether (e.g. northern Hillbrow) indicating more than inability to sketch or faulty memory and indicate rather an emotionally negative response to those areas.

## Conclusions

There is growing recognition of the importance of qualitative research methods in clinical trial research [27] despite the perceived burden this poses to research sites [4]. Ideally, clinical trials should include social research components in the pre-trial stage and throughout the trial itself [28], although this is not always possible due to the massive investment trials require. The local context and what constitutes the trial setting as well as local social divisions and categories, can be critical in understanding the ebb and flow of recruitment and participation in a clinical trial. Cognitive mapping offers a method that could easily be added to the method mix utilised in this type of research.

## Competing interests

The authors declare that they have no competing interests.

## Authors’ contributions

JS conceived the study, assisted in data collection and analysis and drafted the manuscript. CD designed the research, assisted in data collection and analysis and contributed to drafting the manuscript. EV contributed to research design, assisted in data collection and analysis and contributed toward the drafting of the manuscript. CM contributed to research design, assisted in data collection and analysis and contributed toward drafting the manuscript. SD contributed to the concept and contributed to drafting the manuscript. All authors read and approved the final manuscript.

## Pre-publication history

The pre-publication history for this paper can be accessed here:

http://www.biomedcentral.com/1471-2288/13/96/prepub
